# Cigar-Smoking-Cessation Interest and Experience among Black Young Adults: A Semi-Structured In-Depth Interview Investigation

**DOI:** 10.3390/ijerph18147309

**Published:** 2021-07-08

**Authors:** Aniruddh Ajith, Aaron Broun, Danielle A. Duarte, Bambi Jewett, Lilianna Phan, Erin L. Mead-Morse, Mignonne C. Guy, Kelvin Choi, Julia Chen-Sankey

**Affiliations:** 1 Division of Intramural Research, National Institute on Minority Health and Health Disparities, Bethesda, MD 20814, USA; Aniruddh.Ajith@nih.gov (A.A.); abroun@gwmail.gwu.edu (A.B.); danielle.duarte@nih.gov (D.A.D.); bambi.jewett@nih.gov (B.J.); lilianna.phan@nih.gov (L.P.); kelvin.choi@nih.gov (K.C.); 2 School of Medicine, University of Connecticut, Farmington, CT 06032, USA; mead@uchc.edu; 3 Department of African American Studies, College of Humanities & Sciences, Virginia Commonwealth University, Richmond, VA 23220, USA; mguy@vcu.edu

**Keywords:** cigar smoking, African American, young adults, cigar-smoking cessation, healthcare provider counseling, health disparities, health equity, minority health, in-depth interviews, qualitative research

## Abstract

Although Black/African American populations have high cigar-smoking prevalence, little is known about cigar-smoking cessation among this group. This study explored the perceptions and experiences of cigar-smoking cessation and assistance received from healthcare providers among forty Black young-adult cigar smokers (ages 21–29). Semi-structured in-depth phone interviews were transcribed and coded. Qualitative data were analyzed by using thematic analysis. Participants mostly smoked cigarillos, large cigars, and blunts. Overall, many regular cigarillo smokers reported interest in quitting eventually, while large-cigar and blunt smokers shared less interest in quitting because they perceived low harm from smoking these products. The reasons for cigar-smoking cessation were health concerns and financial constraints. Most of the participants who attempted to quit cigars did not use any cessation aids. The reasons for relapse included nicotine withdrawal, stress, and easy access. Additionally, most participants reported their healthcare providers did not ask whether they smoked cigars, and even when they knew, little assistance for cigar-smoking cessation was provided. Informing Black cigar smokers of the harm of cigar smoking and encouraging healthcare providers to screen for and assist with cigar-smoking cessation may alleviate the health burden of cigar smoking in this population.

## 1. Introduction

Non-Hispanic Black/African American adults in the US experienced a significant increase in cigar-smoking prevalence between 2002 and 2016, while the prevalence among other racial/ethnic adults remained stable [1]. This trend threatens to exacerbate existing tobacco-related health disparities for Black adults, as cigar smoking is associated with detrimental health outcomes, including lung, oral, and esophageal cancers [2]. The health risks of smoking cigars vary by cigar type (e.g., large cigars, cigarillos, and filtered cigars) [3], and some cigar types can be more detrimental to health than cigarettes [3]. For example, cigarillos or little cigars, the most commonly used types of cigar products in the US, can contain a greater amount of tobacco and more carcinogens per gram than cigarettes [4], and they may promote nicotine dependence and expose users to a considerable amount of carbon monoxide [5]. Large or premium cigars can also deliver 10 times the nicotine, two times the tar, and five times the carbon monoxide than cigarettes [3]. Although not all cigar smokers inhale while smoking, many of them inhale cigar smoke just like cigarette smoke, potentially exposing themselves to substantial amounts of nicotine and harmful chemicals [2]. Therefore, using these cigar products is likely to pose significant short-term and long-term health risks. Additionally, cigars are often smoked as blunts, whereby part of the tobacco is hollowed out to be replaced with cannabis. Smoking cannabis with tobacco products exposes users to the same tobacco carcinogens, suggesting that blunt smoking may also be a risk factor for cancer [6]. Further, long-term cannabis use is associated with memory impairments and attention deficits [7], and concurrent use of tobacco and cannabis increases symptoms of cannabis dependence [8].

Cigar products are disproportionately smoked by Black individuals [9,10,11] and young adults [12,13]. Increased cigar smoking is a consequence of the tobacco industry’s targeted marketing strategies for selling and promoting cigar products among Black communities [14]. Nationally representative data from 2014 to 2015 showed that, compared to non-Hispanic White adults, non-Hispanic Black adults were more likely to smoke cigar products in the past 30 days (13.8% vs. 5.9%), be daily cigar smokers (1.9% vs. 0.5%), and have smoked cigars “fairly regularly” before (5.4% vs. 2.5%) [10]. Additionally, more than half of non-Hispanic Black adults who currently smoked cigars had smoked blunts in the past 12 months as compared to about a quarter of non-Hispanic White cigar smokers [10]. Given the high prevalence of cigar smoking and co-using with cannabis among non-Hispanic Black adults, supporting this population with cigar-smoking-cessation initiatives is crucial to preventing and reducing cigar-associated morbidity and mortality in the US.

Previous research on cigar smoking among the Black population has mainly focused on the patterns and risk factors of cigar initiation and current use [1,10,12,15]. Few studies, however, have examined Black cigar smokers’ experiences with quitting cigar products. Furthermore, little is known about the role of healthcare providers in assisting Black cigar smokers with cigar-smoking cessation. Healthcare-provider counseling has proven to be an effective strategy to promote cigarette-smoking cessation and has been utilized broadly in primary healthcare settings [16,17]. Hence, research on understanding Black cigar smokers’ perceptions of and experiences with cigar-smoking cessation, as well as their experience with receiving assistance for cigar-smoking cessation from healthcare providers, is critically needed to inform evidence-based approaches that may promote cigar-smoking cessation among this group.

Therefore, by using semi-structured in-depth interviews, this study explored the topics related to the perceptions of and experiences with cigar-smoking cessation and the assistance received from healthcare providers for cigar-smoking cessation among Black young-adult cigar smokers. Young-adult cigar smokers, instead of older smokers, were the target population for this study not only because young adults have higher cigar-smoking prevalence than older adults [18], but also because there are substantial benefits of tobacco-use cessation during young adulthood, prior to developing severe health consequences of long-term tobacco use [19,20,21].

## 2. Materials and Methods

### 2.1. Participant Recruitment and Study Procedures

We conducted telephone-based semi-structured, in-depth interviews with 40 non-Hispanic Black young-adult cigar smokers. Participants were recruited through online social media posts (Instagram, Craigslist, and Facebook). Most of the participants resided in the Washington, D.C., area. Eligibility criteria were (1) self-identifying as non-Hispanic African American or non-Hispanic Black; (2) 21 to 29 years old; (3) being a current cigar smoker, defined as smoking large cigars, cigarillos, or filtered cigars at least 4 times in the past 2 weeks [9,22]; and (4) able to read and speak English. Participants were screened through a self-administered eligibility screening survey online, followed by a brief phone call to further confirm eligibility. Eligible participants were then sent an email with a link to an online survey through which they provided informed consent and information on their sociodemographic characteristics (e.g., age, biological sex, education level, etc.) and tobacco use history (e.g., past and current use of various tobacco products, including cigars, cigarettes, e-cigarettes, etc.). Participants who provided informed consent and completed the online survey were then contacted by research staff to schedule phone interviews. Participants received $100 Amazon gift cards once they completed the phone interview.

### 2.2. In-Depth Interview Procedure

An experienced qualitative researcher served as the interviewer for this study. In-depth interviews have been established as an honest, effective method for focusing on interviewees’ perspectives and experiences [23]. A semi-structured format allows the researchers to ask open-ended questions to enable the interviewers to comprehensively and effectively explore the relevant topics [24,25]. The phone interviews were audio-recorded with consent from participants. A semi-structured interview guide was used to gauge participants’ most frequently smoked cigar products, interest in and perceptions of quitting cigar products, experience with quitting cigar products, and experience with receiving cigar-smoking-cessation assistance from their healthcare providers. Notes were taken by the interviewer during each interview session. Interviews lasted between 45 and 60 min and were conducted between 15 May 2020 and 29 June 2020.

### 2.3. Data Coding and Analysis

Participants’ sociodemographic characteristics and tobacco use history were summarized using Stata 16.0 (StataCorp LLC, College Station, TX, USA) by calculating the percentages of categorical variables as well as the average and the standard deviation of the numerical variables. In-depth interviews were transcribed verbatim from audio recordings, and the transcripts were imported into Dedoose^®^ (Los Angeles, CA, USA), a web-based platform for organizing and coding qualitative data. Four research team members used an iterative process to develop codes based on topics of interest (i.e., interest in and perceptions of cigar-smoking cessation, experience of quitting cigars, and healthcare provider assistance on cigar-smoking cessation) and emerging content from the interview data. Codes were further categorized for their relevance to four specific cigar product types: large cigars, cigarillos, filtered cigars, and blunts. Upon finalizing the codebook, each transcript was independently coded by two trained coders. Coders then met with the entire research team to reconcile any coding disagreements. Satisfactory inter-coder reliability was achieved with a percentage agreement of 82–88% for all the codes used in the study [26].

Thematic analysis was used to analyze coded data generated from the interviews [27,28]. Specifically, two members from the research team independently reviewed all the coded data to generate themed content corresponding with the a priori themes and newly emerged themes. Two members then met to discuss the themes that they generated independently. These themes were then further evaluated and reviewed by the coders who were familiar with the content of the transcripts. Finally, any disagreements with the themes were discussed and reconciled during the meeting with the entire research team before reaching an agreement on the final set of themes to be reported in the current manuscript. The research team also agreed that qualitative data saturation was reached at interview #30. An estimated frequency of participants reporting themes was recorded and reported as “all” (100%), “most” (80–99%), “majority” (60–79%), “more than half” (51–59%), “half” (50%), “less than half” (30–49%), “some” (11–29%), and “a few” (1–10%) [29,30]. The themes were organized in Table 2 by estimated frequency. Based on the interview data, participants were characterized by the most frequent cigar product (large cigars, cigarillos, filtered cigars, and blunts) they currently smoked. The themes specific to filtered cigar smoking and cessation were not reported in the current manuscript due to the lack of interview data specifically related to filtered cigar smoking and cessation. Represented quotes from the participants are chosen for each theme and subtheme. The quotes are labeled by participants’ biological sex, age, and their frequently smoked cigar product type.

## 3. Results

### 3.1. Participant Characteristics

Participants’ mean age was 26 years old (SD = 1.5), and slightly more than half of the participants were female (n = 23, 57.5%) (Table 1). Thirty-six (90.0%) participants reported past-30-day smoking of cigarillos, 24 (60.0%) large cigars, 23 (57.5%) blunts, and 7 (17.5%) filtered cigars. Eighteen (45.0%) participants most frequently smoked blunts, 16 (40.0%) most frequently smoked cigarillos, 4 (10.0%) most frequently smoked large cigars, and 2 (5.0%) most frequently smoked filtered cigars. Additionally, 23 participants (57.5%) reported past-30-day use of cigarettes, 26 (65.0%) reported e-cigarettes, and 27 (67.5%) reported hookah.

### 3.2. Interest in Quitting Cigar Products

When participants were asked about their interest in quitting the cigar products that they currently smoked, most reported low or no interest in immediately quitting (Table 2). Participants mentioned they were not interested in quitting soon mainly because they were not ready to give up cigar or blunt smoking, or because they enjoyed the stress-relieving effects from smoking these products. Participants’ level of interest and reasons for interest in quitting cigars in the future varied by the type of cigar product they most frequently smoked. Almost all frequent cigarillo smokers were interested in quitting sometime in the future, citing avoiding adverse health outcomes as a reason (e.g., upper respiratory problems, sinus and taste problems, and deteriorating cognitive outcomes such as impaired memory). Other reasons included wanting to live a healthy lifestyle, creating a healthy, smoke free environment for their children, and saving money, especially if cigarillos become more expensive and/or if they lose their source of income.

*I don’t think I was in the space of mind to quit yet. [I was not] in a good place. I don’t know how people feel when they quit, but I wasn’t ready. Maybe in the future, I’ll try again.*

*(Woman, 28, cigarillos)*

*Maybe within the next two years or so. I am becoming more health-conscious and aware and I just don’t think [cigarillos] are very sustainable for long-term use, especially if I want to start living a healthier lifestyle.*

*(Woman, 25, cigarillos)*

*I don’t see myself smoking this consistently, but for now, just taking advantage of my situation, my age, not really having to answer to anyone and these are hard times, so I feel like it’s allowed at the moment. Five years from now, no.*

*(Man, 28, cigarillos)*

*I don’t see it as a problem because I don’t see it affecting me. I don’t see myself stopping unless maybe a health risk may present itself. Other than that, I don’t see myself quitting any time soon.*

*(Man, 27, cigarillos)*

The majority of participants who most frequently smoked large cigars expressed much less interest in quitting in the future because they perceived that smoking large cigars did not affect their health or finances. Most of these participants considered smoking large cigars to be a relaxing and enjoyable activity rather than an addictive behavior. Some mentioned that they only smoked large cigars occasionally and/or in social settings with friends and family.

*I only smoke them when I visit my dad and uncle. I don’t think it will do any harm even in the long run.*

*(Woman, 23, large cigars)*

*If I really want to, I can stop. When I want to I will. I will miss going to the lounge because there are so many cool, unique people there. Different ages. Cool environment. I’ll get to the point where I want to stop one day.*

*(Woman, 29, large cigars)*

Similarly, most blunt smokers reported low or no interest in quitting blunts in the future, stating that they did not consider smoking cannabis harmful, or at least as harmful as smoking tobacco, and that they greatly valued the stress relief, pain relief, and relaxation effects of smoking blunts. A few also mentioned that they smoked blunts to cope with negative affect experienced in daily life. Among the few blunt smokers who expressed interest in quitting blunts in the future, they reported avoiding adverse, unexpected side effects of cannabis use and securing a negative drug test for better employment opportunities as potential reasons for quitting. Some regular blunt smokers said that quitting blunts meant quitting cigarillos, and they would find a “healthier” or “safer” alternative method to consume cannabis, such as using rolling papers.

*I’m still not going to stop smoking. I remember I had a panic attack, and the first thing I did was smoke.*

*(Man, 25, blunts)*

*The blunts with weed are going to stay for life. Blunts get you high. Tobacco just relaxes you. I want to be high.*

*(Woman, 27, blunts)*

*I feel like I just need to smoke weed and smoke it out of a bowl, or something like that. Something that’s not tobacco because I know tobacco can be addicting, if I let it be.*

*(Woman, 23, blunts)*

*I’ll probably stop smoking cigarillos, but as far as marijuana, until I figure out my pain situation, I probably will continue to smoke weed.*

*(Woman, 29, blunts)*

*When I say that I think about quitting cigarillos, I’m just talking about quitting the actual cigarillo itself and maybe switching to use hemp papers or papers to smoke marijuana versus using the cigarillos, the tobacco leaves or the tobacco products to smoke it.*

*(Man, 21, blunts)*

### 3.3. Experience of Quitting Cigars and Reasons for Quitting and Relapse

#### 3.3.1. Experience of Quitting Cigars

Despite reports of low interest in quitting cigar and blunt smoking, the majority of participants had tried to quit smoking cigars in the past. Among those who tried to quit, durations of quit attempts ranged from a few days to three months, with most attempts lasting one to two weeks. For quitting large cigars and cigarillos, the most commonly reported quitting method was unassisted through either quitting abruptly (i.e., “cold turkey”) or slowly cutting back (i.e., gradual cessation). A few cigarillo smokers reported using assistance products to quit, such as using nicotine replacement therapy (e.g., nicotine patches). They also reported chewing regular gum and replacing smoking habits with healthier ones such as engaging in physical activity and meditation. A few participants explicitly mentioned that they did not try nicotine replacement therapies because they were unsure whether these methods could be used for cigar-smoking cessation.

*The first day [of cutting back] I [smoked one cigarillo], and then I did cold turkey and it was not fun.*

*(Woman, 28, cigarillos)*

*What I tried to do was quit to a minimum, but it didn’t work. Each time I smoke, I want to smoke more.*

*(Man, 28, large cigars)*

*I had chewing gum. It was fun to chew gum. It helped a lot. It gave me something to do. And it took my mind off [smoking]. Chewing gum or eating chips, or crackers, or doing something with your fingers is a part of the stimulation. It’s the stimulation with chewing and having something to fiddle with or to keep your mind occupied.*

*(Man, 26, cigarillos)*

*I would just try to [quit cigars] cold turkey. I’ve heard about [nicotine patches and gum], but I’ve never thought about them because I don’t smoke cigarettes.*

*(Man, 27, cigarillos)*

Most blunt smokers who reported past quit attempts mentioned that they tried to quit without using any assistance; some reported that they switched to rolling papers for smoking cannabis instead of blunts. Some participants shared that they were unable to find a pharmacological method to quit cannabis and thought that nicotine replacement therapies would not work for quitting blunts because they mainly craved cannabis, not tobacco.

*I don’t know any other way to stop smoking [cannabis] other than doing cold turkey. We don’t have Chantix like for cigarette smokers. There isn’t any protocol to stop smoking weed other than just stop doing it.*

*(Woman, 28, blunts)*

*The older I get the more health conscious I get so it’ll probably transfer over completely to just rolling papers.*

*(Man, 27, blunts)*

#### 3.3.2. Reasons for Quitting Cigars

Among those who have tried to quit cigar products in the past, health concerns from smoking cigars and financial pressures were the most common reasons for quitting. For those who had tried to quit blunts, the primary motivator was improved employment prospects. A few current poly-tobacco product users considered cigarettes to be more harmful than cigars and prioritized quitting cigarette smoking over cigar smoking. Hence, they mentioned trying to use e-cigarettes to quit cigarettes, but continued using cigar products. Two participants also explicitly mentioned that they preferred cigars over e-cigarettes because they enjoyed cigars’ natural tobacco taste.

*For blunts, it more so like I was looking for a job, so I have to take drug tests and such.*

*(Woman, 29, blunts)*

*Buying [cigarillos] every day, it’s expensive.*

*(Woman, 25, cigarillos)*

*I smoked a blunt or whatever and I was in the shower, and I just felt my mouth was a mad dry, cottonmouth. That was the first time I ever heard of cottonmouth and that […] ever happened to me. My neighbor had to take me to the hospital. I didn’t want any more of it. I took a break after that.*

*(Man, 29, blunts)*

*Because I lose my memory really badly. When I’m high, or when I smoke [cigarillos], I can’t even think straight.*

*(Woman, 23, blunts)*

*Large cigars have a smoother taste [compared to e-cigarettes]. It doesn’t taste manufactured.*

*(Man, 27, large cigars)*

#### 3.3.3. Reasons for Cigar-Smoking Relapse

Participants who had tried to quit cigars and blunts reported various reasons for relapse, including a lack of interest in quitting cigars, missing the relaxing effect and rush of smoking, and easy access to affordable cigar products. Participants who frequently smoked cigarillos or blunts and reported reasons for relapse mentioned that without smoking cigarillos or blunts, they had difficulty relieving stress when facing anxiety-inducing situations or coping with negative affect in daily life. Some also discussed that the ample and easy accessibility to cigar products and high affordability of the products triggered smoking relapses. One participant chewed regular gum to help quit cigarillo smoking but relapsed because the gum was more expensive than cigarillos. A few participants also reported that they were trying to quit cigar products prior to the COVID-19 pandemic, however, due to the increased stress and anxiety experienced during the pandemic, they relapsed.

*I feel like something was missing. I needed that extra boost, and that’s what made me want to go back to [cigarillos]. Wanting that extra little buzz and adrenaline rush. I went back to it. It felt empty after a while. Sometimes not doing it can feel a little empty like something’s missing.*

*(Man, 29, cigarillos)*

*Yes, stress. We didn’t know if [aunt] was going to live. I was on pins and needles so I went back [to smoking].*

*(Woman, 28, cigarillos)*

*I stopped chewing [regular] gum. I’d rather smoke those Black & Mild. Gum was expensive, $1.69 really.*

*(Man, 26, cigarillos)*

*I didn’t really fully want to stop. I still have access to it. That’s why I didn’t stop. I tried. I swore I was, but no, it didn’t happen. It lasted all of two days.*

*(Woman, 27, blunts)*

### 3.4. Healthcare Providers Assistance for Quitting Cigar Products

#### 3.4.1. Lack of Cigar-Smoking-Cessation Assistance from Healthcare Providers

Less than half of the participants reported that their healthcare providers knew about their cigar-smoking behavior. They reported that their healthcare providers learned it through initial screeners or checklists at their wellness visit. Participants stated that these screeners usually asked about general tobacco use but not about the specific type of tobacco product used. Among participants who reported current tobacco use on the screener, the majority reported that their healthcare providers did not ask follow-up questions about their tobacco use or clarify what type of tobacco product(s) they used. Only two participants reported that their healthcare providers knew the type(s) of tobacco product they currently used. A few participants noted that their healthcare providers followed up with questions about their overall tobacco use behavior by briefly discussing the harm of smoking and handing out brochures with cessation information.

*That’s not the first doctor that has not asked me about it like I don’t think that is a common practice unless you have lung issues or something that is specifically tied to smoking. I don’t think doctors would ask you about it.*

*(Woman, 28, blunts)*

*I told them that I have smoked before, but that’s it. They ask you if you smoke, that’s it.*

*(Woman, 25, cigarillos)*

*My doctor asked me if I smoke, but she never said, “Would you like to stop?” Or anything like that. It was just on the questionnaire and she asked questions about it, I answered it and we just moved on.*

*(Woman, 28, blunts)*

*No. My doctor here…we have about 15 min, and he asks me do I have any problems, issues and then he just sends me on my way.*

*(Man, 27, cigarillos)*

*They ask me when I go, “Do you smoke?” I say yes, and they give me basically the reasons why I shouldn’t smoke and basically inform me about it.*

*(Man, 22, blunts)*

Among those who received information about the harm of cigar smoking, some stated that this information was not effective for them to consider stopping smoking. One participant explicitly recalled that their healthcare provider suggested not to smoke cigars heavily, but “in moderation.” When asked by the interviewer, only two participants reported that their healthcare providers spent time discussing methods and options for smoking cessation during the healthcare visit. Moreover, these conversations were on smoking cessation in general rather than quitting cigar or blunt products specifically. Two participants mentioned that they would consider quitting cigars if their healthcare providers asked them to quit.

*Yes, I fill it out on the paperwork. I ask them, and they’re like, “You shouldn’t smoke cigars but if you’re going to do it, do it in moderation”.*

*(Woman, 28, cigarillos)*

*I think if the doctor said, “You need to quit. You have this condition and if you don’t…” like that, it would probably have to be something affecting my health that would make me say, “You need to stop”.*

*(Woman, 28, cigarillos)*

*I haven’t really had a full conversation about it. They ask me when I go, “Do you smoke?” I say yes, and they give me basically the reasons why I shouldn’t smoke and basically inform me about it, and at that time I’ll be like, “Okay,” and that’s pretty much it.*

*(Man, 22, blunts)*

#### 3.4.2. Interest in Discussing Cigar and Blunt Smoking with Healthcare Providers

The majority of large cigar and cigarillo smokers reported that they were willing to discuss cigar-smoking behavior and cessation with their healthcare providers, especially if their healthcare providers asked them questions and assessed their interest in quitting cigars. Many of them also expressed interest in learning from their healthcare providers about the ways to quit cigar smoking. Some blunt smokers were also willing to discuss blunt smoking behaviors with healthcare providers and were eager to learn about health harms of smoking blunts and cannabis.

*I don’t know how [smoking blunts] really affects my health. Maybe if they could provide me with statistics like people that smoke marijuana or smoke blunts are more likely to….*

*(Man, 23, blunts)*

*I have the conversation with my doctor about the risks of smoking, the things that can happen if I continue to do this lifestyle. Those are just conversations that I don’t really want to think about right now. I’ll think about those things maybe when I’m over in my 30s, my 40s.*

*(Woman, 29, cigarillos)*

Participants who reported no interest in discussing cigar smoking with their healthcare providers mentioned that they already knew the negative health consequences of smoking cigars, they did not trust their healthcare providers, or they wanted to make their own health decisions. These participants expressed that they intentionally checked “No” to the questions on the tobacco screener to avoid having a conversation with their healthcare providers about their tobacco use. Many regular blunt smokers mentioned that they would not discuss blunt smoking or quitting blunts with their healthcare providers because they were not interested in quitting cannabis and/or they did not want their healthcare providers to know they were using cannabis.

*They never ask me, because I’m pretty sure if I would put “yes,” it would be like, “You mentioned that you smoke marijuana.” I would check “no.” For confidential reasons, I don’t want my doctor knowing that I engage in those activities.*

*(Woman, 23, blunts)*

*I didn’t want to tell them that I was smoking because I know what the side effects of smoking. I fully understand what they are, and I don’t like to be told what they are.*

*(Man, 25, cigarillos)*

*I would if it was an issue to me but because it’s not and I know the stigma that surrounds it. I don’t think that I will want my doctor to feel any negative or positive way about me because of it. I wouldn’t volunteer that information unless it was something that was specifically important to ask during my doctor’s visit.*

*(Woman, 28, blunts)*

## 4. Discussion

This is one of the first studies to investigate topics related to cigar-smoking cessation among Black young adults, a population with one of the highest cigar-smoking prevalence rates of all groups in the US [1,10]. Results from this semi-structured in-depth interview study suggests that Black young-adult cigar smokers may have low interest in quitting smoking any type of cigar product in the near future. Common reasons for cigar-smoking relapse after quitting attempts were nicotine withdrawal, smoking to cope with stress, and easy accessibility to cigar products. Moreover, participants reported limited discussion and assistance for cigar-smoking cessation from their healthcare providers. Evidence from this study can inform public health programs and healthcare practices in promoting and improving cigar-smoking cessation among Black young-adult cigar smokers.

The lack of immediate interest in quitting any type of cigar product among Black young-adult cigar smokers in this study is especially concerning given that cigar products can contain a comparable or higher amount of nicotine and carcinogens than conventional cigarettes [4,5]. Positive expectancies of smoking large cigars for social purposes and smoking blunts for stress relief may explain participants’ low interest in quitting these products, even in the distant future. Low motivation to quit cigar products may also be influenced by young adults’ perceptions of low risk and harm from cigar smoking, a finding which is supported by previous research [13,31]. The lack of interest in quitting blunt products, even in the long-term, may also be explained by the low risk and harm perceptions of cannabis use among young adults [32,33,34]. This low interest in quitting cigar and blunt products may promote continued tobacco and cannabis use and dual use among Black young adults, exacerbating the health disparities from using these harmful and addictive products. As state- and local-level cannabis legalization advances in the country, more research is needed to understand whether and how this policy development may influence blunt smoking and blunt-smoking cessation. Increasing harm perceptions about cigar and cannabis smoking may motivate cigar smokers’ immediate or long-term interest in quitting these products.

The current study also suggests that Black young-adult cigar smokers were unassisted for most of their attempts to quit cigars, particularly without using evidence-based cessation therapies. This finding may be due in part to participants’ uncertainty with whether proven smoking-cessation therapies were effective for quitting cigar products. Clinical trials have shown that behavioral programs and pharmacotherapies are effective for cigarette-smoking cessation, and a few studies have examined smokeless tobacco cessation [35]. However, no studies to date have examined their efficacy for smoking cessation of cigar products. Moreover, this study showed that the reasons for cigar-smoking relapse were similar to those for cigarette-smoking relapse, including a lack of coping mechanisms for stress and anxiety as well as nicotine cravings and withdrawal from nicotine addiction [35]. Therefore, behavioral therapies and pharmacotherapies proven to be effective for cigarette-smoking cessation hold promise for increasing cessation and reducing relapse of cigar smoking. Future research is needed to understand the efficacy of these smoking-cessation therapies on cigar-smoking cessation in the general population and particularly among Black cigar smokers. If proven effective, these programs should be promoted among Black young-adult cigar smokers in community and clinical settings.

The study results also suggest that Black young adults in our study perceive a lack of assistance in cigar-smoking cessation from their healthcare providers. Previous research has shown that cigarette smokers who received smoking-cessation counseling from their healthcare providers were more likely to quit smoking as compared to those who did not receive counseling [16,17]. However, only a few participants in our study reported that their healthcare providers asked about their tobacco use status and many did not ask about the use of specific tobacco products. Moreover, many healthcare providers did not ask follow-up questions when participants reported that they currently smoked tobacco or cigars. These results are consistent with some previous research showing that tobacco-smoking-cessation counseling is underutilized by healthcare providers [36,37] and that healthcare providers are less likely to conduct screening and administer smoking-cessation intervention for non-cigarette tobacco users [38]. The healthcare providers may help improve the motivation and outcome of cigar-smoking cessation by screening patients for the use of cigar products, discussing the potential harm of cigar smoking with the patients, encouraging the patients to create cigar-specific smoking-cessation plans, and referring patients for cigar-specific cessation counseling.

Besides the lack of proven therapies and services for cigar-smoking cessation and the underutilization of cigar-smoking-cessation counseling by healthcare providers, Black cigar smokers may potentially face additional barriers in quitting cigar products compared to White cigar smokers. Studies of cigarette-smoking cessation have already shown that, compared to their White counterparts, Black smokers are less likely to use pharmacotherapies for quitting cigarettes, potentially due to the lack of knowledge about and affordable access to the therapies [39,40]. Black smokers also experience higher levels of smoking relapse triggered by heightened stress and anxiety induced by racial/ethnic social discrimination and injustices [41]. Additionally, the affordability of and abundant access to cigar products have been frequently found among communities with high concentration of Black populations, especially in urban and suburban settings [42,43,44]. This easy accessibility to cigar products may trigger smoking relapses among Black cigar smokers when they attempt to quit cigars, similar to what has been found for cigarette-smoking cessation [14,45,46]. Healthcare providers and public health practitioners should implement culturally and contextually tailored cigar-smoking-cessation assistance and interventions for Black cigar smokers, as these tailored programs may help further improve smokers’ interest and outcomes in quitting cigars products.

Although our participants expressed little motivation for quitting blunts, a few of them expressed interest in learning more about the harm and long-term effects of using cannabis and the methods for quitting cannabis. Some participants also realized that smoking blunts might not be the safest method to consume cannabis and expressed interest in learning about non-combustible methods of consuming cannabis. Healthcare providers and public health practitioners should provide education about the health risks of cannabis use and the methods for consuming cannabis safely. It may also be beneficial to increase harm perceptions of cannabis use among Black young adults by emphasizing the long-term cognitive and physical health consequences of using the product and the exacerbated health outcomes of concurrently using cannabis and tobacco [7,8]. As many Black young adults used cigars for smoking blunts, more research is needed to investigate how blunt smoking behavior may influence cigar-smoking-cessation motivation and outcomes among this population.

The current study has the following limitations. First, while the sample was purposeful to qualitatively assess cigar-smoking-cessation experiences among Black young-adult cigar smokers, findings may not be representative of Black young-adult cigar smokers living in other areas of the country where cigar and cannabis use patterns and policies were different. Second, our study examined participants’ perspectives of cigar-smoking-cessation assistance from their healthcare providers. Future research should examine healthcare providers’ perspectives on the topic to further understand the barriers and facilitators of providing cigar-smoking-cessation assistance and counseling to patients. Third, the data were collected during the beginning of the COVID-19 pandemic when participants experienced heightened stress and anxiety [47]. This might have contributed to low interest in cigar-smoking cessation in the near term since cigars and blunts are reportedly used to relieve stress and cope with negative affect.

Regardless of the limitations, this is one of the first studies to explore the perceptions and interests in cigar-smoking cessation among Black young adults. More research is needed to understand the social and behavioral predictors of cigar-smoking cessation and how assistance from healthcare providers may improve cigar-smoking-cessation interests and outcomes among Black young adults.

## 5. Conclusions

This is one of the first studies to explore the perceptions and experiences of cigar-smoking cessation among Black young-adult cigar smokers. Considering the high cigar-smoking prevalence among Black young adults, the promotion of cigar-smoking cessation is especially important in this group to reduce health disparities and health effects associated with cigar smoking. Results from this study indicate the importance of increasing harm and risk perceptions among Black cigar smokers in order to promote their interest in cigar-smoking cessation. The results also highlight the need to improve healthcare practices for assisting Black young-adult cigar smokers in quitting cigar products.

## Figures and Tables

**Table 1:** Participant characteristics (n = 40).

	Mean	SD
Age		
	26.0	2.4
	**n**	**%**
Biological Sex		
Male	17	42.5%
Female	23	57.5%
Education Level		
≤GED or high school	7	17.5%
Some or completed technical school	9	22.5%
Some college	15	37.5%
≥Bachelor’s degree	9	22.5%
Current Employment Status		
Full time	19	47.5%
Part time	7	17.5%
Unemployed	11	27.5%
Others	3	7.5%
Current Financial Situation		
Live comfortably	13	32.5%
Meet needs with a little left	15	37.5%
Just meet basic expenses	12	30.0%
Cigar Smoking in the Past 30 Days		
Large cigars	24	60.0%
Cigarillos	36	90.0%
Filtered cigars	7	17.5%
Blunts	23	57.5%
Number of Cigar Products Smoked in the Past 30 Days		
One product	4	10.0%
Two products	16	40.0%
Three products	11	27.5%
Four products	9	22.5%
Most Frequently Smoked Cigar Product in the Past 30 Days		
Large cigars	4	10.0%
Cigarillos	16	40.0%
Filtered cigars	2	5.0%
Blunts	18	45.0%
Use of Other Tobacco Products in the Past 30 Days		
Cigarettes	23	57.5%
E-cigarettes	26	65.0%
Hookah	27	67.5%

**Table 2:** Reported themes by estimated frequencies.

Themes	Frequency
Interested in quitting cigar products	
Yes	Some
No	Most
Previous cigar quit attempts	
Yes	Majority
No	Less than half
Healthcare providers’ awareness that participants smoked cigars	
Yes	Less than half
No	Majority
